# Cancer-Specific Telomerase Reverse Transcriptase (TERT) Promoter Mutations: Biological and Clinical Implications

**DOI:** 10.3390/genes7070038

**Published:** 2016-07-18

**Authors:** Tiantian Liu, Xiaotian Yuan, Dawei Xu

**Affiliations:** 1 Department of Pathology, Shandong University School of Medicine, Jinan 250012, China; 2 Department of Medicine, Division of Hematology and Center for Molecular Medicine (CMM), Karolinska Institutet and Karolinska University Hospital Solna, Stockholm SE-171 76, Sweden; xiaotian.yan@ki.se; 3 Shandong University-Karolinska Institutet Collaborative Laboratory for Cancer Research, and Central Research Laboratory, Shandong University Second Hospital, Jinan 250033, China

**Keywords:** cancer, cancer diagnosis, cancer prognosis, telomerase, TERT promoter mutations

## Abstract

The accumulated evidence has pointed to a key role of telomerase in carcinogenesis. As a RNA-dependent DNA polymerase, telomerase synthesizes telomeric DNA at the end of linear chromosomes, and attenuates or prevents telomere erosion associated with cell divisions. By lengthening telomeres, telomerase extends cellular life-span or even induces immortalization. Consistent with its functional activity, telomerase is silent in most human normal somatic cells while active only in germ-line, stem and other highly proliferative cells. In contrast, telomerase activation widely occurs in human cancer and the enzymatic activity is detectable in up to 90% of malignancies. Recently, hotspot point mutations in the regulatory region of the *telomerase reverse transcriptase (TERT)* gene, encoding the core catalytic component of telomerase, was identified as a novel mechanism to activate telomerase in cancer. This review discusses the cancer-specific TERT promoter mutations and potential biological and clinical significances.

## 1. Introduction

Telomerase is a RNA-dependent DNA polymerase lengthening telomeric DNA (TTAGGG repeats) at the termini of chromosomes [1,2]. Most normal human somatic cells lack telomerase activity due to the tight transcriptional repression of its rate-limiting, catalytic component *telomerase reverse transcriptase (hTERT)* gene [1,2,3]. This, together with the end replication problem, leads to progressive telomere shortening resulting from cellular replication. When telomere shortens to a critical size (dysfunctional), cells are triggered to enter a permanent growth arrest stage called senescence [1,2,4]. Therefore, telomere erosion-mediated cellular senescence confers normal cells a limited life-span. In contrast, infinite proliferation is a hallmark of malignant cells [1,2]. Conceivably, overcoming senescence barrier by telomere stabilization is required to acquire infinite cell proliferation potential in oncogenesis, and, in most cases, this is achieved by transcriptional induction of TERT expression accompanied by telomerase activation [1,2]. In accordance, TERT expression and telomerase activity is widespread and detectable in the majority (up to 90%) of human malignancies [1,2,4].

Given the key role of telomerase or TERT in malignant transformation, great efforts have been made to dissect mechanisms underlying telomerase activation and TERT induction. Recently, hotspot TERT promoter mutations were identified to stimulate the TERT transcription or telomerase activation and to occur in various types of cancer [5,6,7]. In the present review, we discuss these new findings and biological, clinical implications of TERT promoter mutations in human malignancies.

## 2. TERT Transcription: Aberrant Activation in Cancer

**Figure 1:**
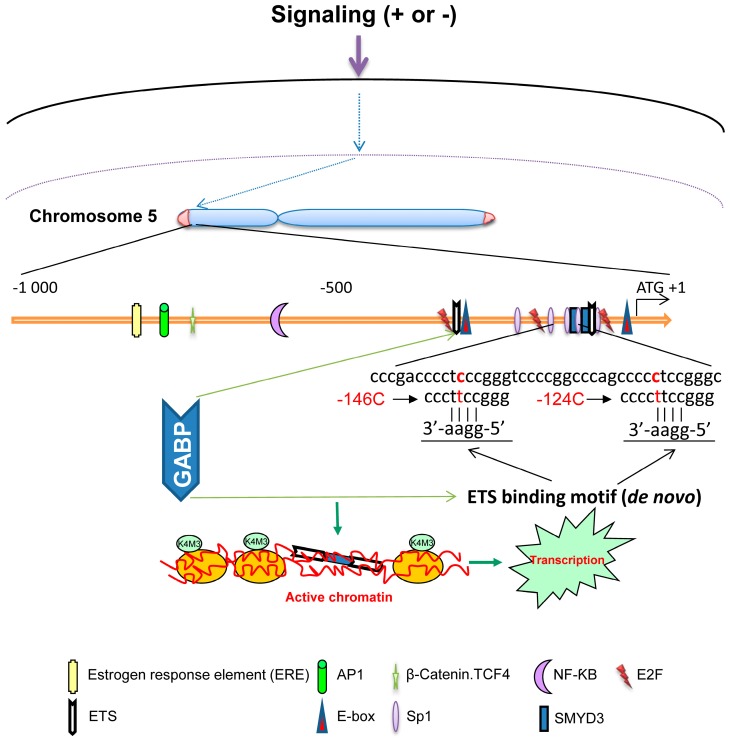
Schematic illustration showing the TERT promoter with its important transcription factor binding sites and cancer-specific TERT promoter mutations. The *TERT* gene is at chromosome 5p and its promoter (from ATG to −1000) with binding motifs for important transcription factors is shown. Both positive and negative regulators for TERT transcription signal to the TERT promoter where transcription factor binding occurs and the local chromatin becomes open or close. In malignant cells, C>T mutation may take place at one of both positions of the TERT proximal promoter (−124 and −146 to ATG for −124C>T and −146C>T, respectively). These mutations create de novo ETS1 binding motifs. The ETS transcription family member GABP is recruited to the promoter and binds both native and de novo ETS motifs as a heterotetrameric complex, which leads to increased histone H3-K4 trimethylation and opened chromatin.

The compelling evidence has accumulated that the *TERT* gene is predominantly governed at transcriptional levels. The transcriptional controlling of the *TERT* gene is extremely complex and includes regulation at multiple levels by various positive and negative factors or pathways [8,9]. These different factors may affect TERT transcription independently or interdependently. The cloning of the TERT promoter and identification of its various transcription factor binding motifs has gained profound insights into the molecular mechanism of TERT and telomerase regulation [10,11,12,13]. The TERT promoter does not have a TATA box region but is GC-rich. This TATA-less promoter harbors at least five upstream Sp1 binding motifs, two E-boxes and a single transcription start site that binds multi-functional transcription factor TFII-I for gene expression (Figure 1) [10,11,12,13]. By interacting with various transcription factors, the TERT promoter responds to numerous signals and integrates those diverse and dynamic inputs to set the TERT mRNA output. Moreover, epigenetic effects on chromatin structure and remodeling of the *TERT* promoter region add another layer controlling to the TERT transcription [14]. In addition, many factors indirectly regulate TERT transcription through interacting with transcription factors or other regulatory elements in a cell-type-dependent manner. All these transcriptional regulators coordinately and tightly control the *TERT* gene to ensure its silence in most normal cells, while its expression at right time, right place and right quantity only in a small subset of cells, such as activated lymphocytes and stem/progenitor cells [2,15].

However, this balance is disrupted in malignant cells, most likely due to aberrant expression of positive regulators or silencing of negative ones. A typical example is the Myc/Max/Mad network proteins, the master regulator of the TERT transcription widely dysregulated in human cancer [16]. In HL60 leukemic cells, high c-Myc expression is coupled with its binding to the E-Boxes on the TERT core promoter and TERT mRNA expression, and once the cells are induced to undergo terminal differentiation, c-Myc expression is diminished whereas Mad1 levels increased and subsequently replace c-MYC on the TERT promoter, thereby silencing TERT transcription [16,17]. Tollefsbol’s group determined the *TERT* gene trans-activation by endogenous c-Myc during the conversion from normal to transformed human fibroblasts, and they found that the induction of c-Myc expression led to a switch from Mad1/Max to c-Myc/Max binding to sequences containing the TERT promoter distal and proximal E-boxes, *TERT* gene and telomerase activation [18]. In addition, many other factors regulate TERT transcription via the Myc/Max/Mad protein family or different mechanisms. These regulators include the TGF-β/Smad signaling pathway, Wnt/β-Catenin, Arsenic, Aurora-A, NFX1 Tax, estrogen, Ets, DJ-1, E2F, survivin, HIFs, FoxM1, Reptin, various growth factors and cytokines, etc. [16,19,20,21,22,23,24,25,26,27,28,29,30,31,32,33,34,35,36].

The presence of transcription factors is critical for the regulation of TERT transcription. However, gene transcription involves not only the assembly of transcription factors at promoter/enhancer regions, but also the regulation of accessibility to DNA, a process controlled by the epigenetic mechanism [16,17,37,38,39,40]. Therefore, epigenetic factors are another group of proteins that actively regulate TERT transcription. It is well established that DNA methylation, histone acetylation, and histone methylation are all involved in the regulation of TERT transcription [16,17,37,38,39,40,41]. The TERT promoter is in general unmethylated in normal cells, while its methylation is required for TERT expression and telomerase activation in cancer cells. Histone acetylation/deacetylation was shown to be a common underlying feature to *TERT* transactivation/repression in both normal and malignant human cells [16,37,38,39]. Mechanistically, transcription factors Myc/Max/Mad and Sp1 interact with and recruit histone acetyltransferases (HATs) or histone deacetylases (HDACs) to the TERT promoter, dependent on the promoter status and cellular contexts [37,38,39]. In addition, SMYD3 as a histone methytransferase is capable of binding to CCCTCC sequences on the TERT promoter and specifically catalyzes H3-K4 tri-methylation, through which TERT transcription is activated. SMYD3-mediated H3-K4 tri-methylation is required for optimal occupancy of c-MYC and Sp1 on the TERT promoter [40]. Therefore, epigenetic factors closely interact and cooperate with transcription factors to exert their effects on TERT transcription.

In addition to the endogenous TERT regulators discussed above, many oncogenic viruses encode proteins that stimulate TERT transcription. These exogenous regulators include Epstein-Barr virus (EBV), Kaposi sarcoma-associated herpesvirus (KSHV), human papillomavirus (HPV), hepatitis B virus (HBV), hepatitis C virus (HCV), cytomegalovirus (hCMV) and human T-cell leukemia virus-1 (HTLV-1) [42,43,44,45,46]. The HPV E6 is the most extensively studied viral oncoprotein for its role in the TERT transcription. E6 forms a tertiary complex with E6AP and c-Myc, and such complex then binds to E-box in the TERT core promoter and subsequently induces the promoter activation [45,46]. The CMV E72 protein robustly activates TERT transcription via Sp1 [44]. Therefore, the targeted activation of TERT transcription is one of the key mechanisms for virus-mediated oncogenesis.

## 3. TERT Promoter Mutations: Novel Mechanism for Telomerase Activation in Malignant Transformation

In 2013, two seminal papers reported recurrent mutations of the TERT core promoter in both sporadic and familiar malignant melanomas [5,6]. The identified hotspot mutations, which cause a cytidine-to-thymidine (C>T) dipyrimidine transition at chromosome 5 1,295,228 and 1,295,250 (−124 and −146 bp from the ATG), are thus named −124C>T and −146C>T, respectively [5] (Figure 1). During last three years, the TERT promoter mutations have been widely investigated and identified in various types of human cancer with different frequencies [5,6,7,47,48,49,50,51,52,53,54,55,56,57,58,59,60,61,62,63,64,65,66,67,68,69,70,71,72,73,74,75,76,77,78,79,80,81,82,83,84,85,86,87,88,89,90,91,92,93,94,95,96,97,98,99,100,101,102,103,104,105,106,107,108,109,110,111,112,113,114,115,116,117,118,119,120,121,122,123,124,125,126,127,128,129,130,131,132,133,134,135,136,137,138,139,140,141,142,143,144,145] (Table 1). Based on these clinical study results, −124C>T mutation is more prevalent than −146C>T among various malignancies.

Both −124C>T and −146C>T mutations have been suggested as oncogenic driver events due to their stimulatory effects on TERT transcription and telomerase activation. Clinically, tumors carrying TERT promoter mutations were frequently observed to express higher levels of TERT mRNA and telomerase activity compared with those having a wt promoter [57,59,122]. Experimentally, the introduction of either −124C>T or −146C>T into the TERT promoter reporter could significantly enhance the promoter activity [5,123]. Chiba et al. created −124C>T or −146C>T mutations of the TERT promoter in human pluripotent stem cells using genome editing, and they found that those cells still expressed TERT and telomerase even after undergoing differentiation [124], in sharp contrast to the wt TERT promoter-bearing stem cell-derived progenies where the *TERT* gene transcription is strictly repressed. Moreover, the differentiated cells with mutant TERT promoter carried longer telomere and erased replication senescence imposed by telomere attrition as seen in normal cells [3,124]. The study thus provides direct evidence that the presence of −124C>T or −146C>T mutation is sufficient to confer cells immortal or sustained proliferation potentials. Mechanistically, −124C>T or −146C>T mutation putatively creates a de novo binding site for ETS transcription factors [5,123]. Bell et al. and Makowski et al. further showed that the multimeric GA-binding protein (GABP), the ETS family transcription factor, was specifically recruited to the mutant rather than wt TERT promoter in different human cancer cells, thereby aberrantly activating TERT transcription and telomerase (Figure 1) [123,125]. In cancer cells carrying heterozygous TERT promoter mutations, the mutant promoter recruits the GABPA transcription factor and exhibits the H3K4me2/3 mark of active chromatin. In contrast, the wild-type allele retains the H3K27me3 mark of epigenetic silencing [126]. These results suggest that only the mutant promoters are transcriptionally active. In addition, CC>TT tandem mutations at position −124/−125bp and −138/−139 were found in a subset of cancer [51,127]. These two tandem mutations also lead to the creation of the ETS transcription factor-binding motif, likely enhancing TERT transcription via similar or identical mechanisms. Taken together, the cooperation between GABP or other ETS family transcription factors and the mutant TERT promoter is a novel mechanism for induction of TERT expression and telomerase activation in cancer.

TERT promoter mutation rates vary significantly from undetectable to 85% among studied human malignancies [79,128]. The mutation occurs most frequently in bladder, renal pelvic, thyroid, hepatocellular cancer, malignant glioblastoma and melanoma [5,6,7,57,79,93,103,122], while it is rarely present in hematological malignancies, prostate, gastrointestinal, breast and lung cancer [7,128,129] (Table 1). It is currently unclear what cause such differential mutation profiles among different types of cancer. It was initially hypothesized that the *TERT* promoter mutation should occur more frequently in tumor types exhibiting high rates of alternating-lengthening of telomere (ALT), however, Killela et al. showed that TERT promoter mutations occur frequently in glioblastomas while rarely in pancreatic neuroendocrine tumors, despite both of them with high rate of ALT [7]. Analyses of thyroid and bladder cancer do not support this hypothesis. Widespread telomerase activation and *TERT* expression has been observed in follicular thyroid cell-derived cancer, and the mutation was observed with a high frequency in these tumors [93,94]. In contrast, 40% of parafollicular C cell-derived medullary thyroid carcinoma (MTC) are negative for *TERT* expression and ALT is utilized to maintain telomere sizes in MTC tumors [130], however, none of these tumors carried the *TERT* promoter mutation [93]. On the other hand, ALT is in general absent in bladder cancer, but up to 85% of bladder cancer harbor TERT promoter mutations [7,79,80]. These results strongly suggest cell type- and origin-dependent *TERT* promoter mutations in cancer. In addition, we as well as others found that the presence of TERT promoter mutations was intimately associated with senior age of patients and shorter telomere in tumors [57,63,93,98], which indicates that these two factors play a part in the mutation event onset during malignant transformation. More recently, Indian patients with cervical cancer were shown to have a high rate of TERT promoter mutations in their tumors [131], in striking contrast to their rarity in this cancer type from other countries [7,132]. Jeon et al. reported rare TERT promoter mutations (<3%) in a cohort of Korea patients with PTC [91,133]. In addition, geographical differences in the mutation rate were observed in hepatocellular carcinoma [101]. Likely, different genetic susceptibility and/or environment exposure may contribute to such disparities in TERT promoter mutations observed in same types of cancer from different geographical areas.

TERT promoter mutations frequently occur together with activating mutation of oncogenic drivers that facilitate cellular replication. For instance, the gain-of function mutation of the *fibroblast growth factor receptor 3* (*FGFR3*) gene is highly prevalent in bladder cancer (BC), and the mutant FGFR3 promotes in BC development by over-stimulating the RAS-mitogen-activated protein kinase (MAPK) and phosphatidylinositide-3 kinase-AKT pathways [77,78,81]. The BRAF^V600E^ mutation, widespread in PTC and melanoma, plays a similar role in these two malignancies. TERT promoter mutations are shown to be tightly associated with the presence of FGFR3 and BRAF^V600E^ mutations in BC and PTC or melanoma, respectively [51,77,78,81,98,99,100,134]. These findings, together with shorter telomere in TERT promoter mutation-positive tumors, support the following model: The onset of TERT promoter mutations results from telomere dysfunction induced by oncogene-mediated cellular over-replication. Further studies are required to dissect a mechanistic link between the mutation and dysfunctional telomeres.

Despite the numerous studies revealing telomerase and *TERT* expression in the vast majority of human cancer, it remains poorly defined when telomerase is activated in carcinogenesis, especially in an in vivo setting. The identification of TERT promoter mutations provides a novel genetic marker to monitor telomerase activation during the process of tumor development. We analyzed TERT promoter mutations in 69 pre-malignant tissue specimens derived from 51 patients with follicular thyroid adenoma (FTA) and 18 with atypical FTA (AFTA) and found four of them with mutations (one from FTA and three from AFTA) [99]. All four of these TERT promoter mutation-carrying samples expressed TERT mRNA and telomerase activity [99]. Consistent with our findings in FTA and AFTA, the TERT promoter mutation was also detected in precursor lesions of hepatocellular tissue [103] and in inverted papillomas and benign urothelial lesions [118]. Earlier reports did not find the TERT promoter mutation in benign nevi [6], while recently detailed analyses of melanoma evolution revealed that the mutation could occur in early stages of melanoma development and be already detectable in 77% of benign nevi, intermediate lesions and melanoma in situ [56]. Collectively, the TERT promoter mutation is an early genetic event in in vivo oncogenesis, which provides the direct evidence that telomerase activation can appear genetically in in vivo carcinogenesis already in “benign” tumors without overt malignant phenotype. Pre-malignant cells harboring TERT promoter mutations are likely more competent to progress to fully transformed cells.

## 4. TERT Promoter Mutations: Novel Biomarkers for Cancer Diagnostics/Screening

Cancer-related TERT expression and telomerase activation is a specific biomarker for malignancies, and efforts have long been made to set up TERT or telomerase detection approaches for cancer diagnosis [80,146,147]. However, a number of issues impede reliable utility of the TERT or telomerase activity assay for diagnostic or screening purpose. First, infiltrated lymphocytes in tumors or exfoliated inflammatory cells have TERT expression, which may cause false-positive results. Second, telomerase and TERT mRNA are highly sensitive to temperature and inappropriate handling, and their quantification needs high-quality tissue samples, which limits their clinical application [80,147]. Finally, commercially available TERT antibodies for immune-histochemical staining or immunoblotting are always problematic with specificity [147]. Given the above drawbacks, it is highly demanding to develop alternative strategies. The presence of TERT promoter mutations in human malignancies whereas their absence in normal tissues/cells provides new cancer-specific markers. Because DNA is sufficient for mutation analyses, its high stability makes the assay easier, especially feasible for routine clinical examination.

Since BC and renal pelvic cancer (RPC) exhibits a high frequency of TERT promoter mutations, the test for them as cancer biomarkers has been predominantly performed on these two malignancies [77,78,80,122,136]. A number of groups tested the mutant TERT promoter detection for diagnostic purpose and recurrence surveillance in urine derived from patients with BC or RPC. We determined the presence of the mutant TERT promoter in urinary DNA derived from BC and RPC patients (collected prior to surgical treatment) using Sanger Sequencing, and 96% specificity with 60% sensitivity was achieved [80,122,136]. One week after surgical treatment, the mutant sequences disappeared rapidly from patient urine. This proof-of-concept study clearly demonstrates usefulness of the mutant TERT promoter as a urinary biomarker for the disease detection/monitoring.

Direct sequencing such as Sanger sequencing is regarded as a gold standard for the identification of mutant targets, however, its threshold sensitivity is at least 10% of mutant TERT promoter-containing tumor DNA. Thus, it is strongly motivated to develop more sensitive assays to detect minor proportions of mutant alleles present in bulk urinary DNA. For this purpose, we recently set up Competitive Allele-Specific TaqMan PCR (castPCR). castPCR could increase the detection limit by four folds (Compared to Sanger Sequencing) and reached 90% of detection sensitivity without compromising specificity [136]. Further optimization of castPCR will certainly improve its detection accuracy and sensitivity. In addition, Liu et al. set up a sensitive Amplification Refractory Mutation System-PCR for the mutation detection with improved sensitivity and specificity [137].

TERT promoter mutations assessed on thyroid fine-needle aspiration biopsy (FNAB) were also evaluated to discriminate between benign and malignant thyroid tumors. Liu et al. analyzed 308 FNAB specimens preoperatively obtained from thyroid nodules and they detected no TERT promoter mutations in 179 benign thyroid nodules while all nine thyroid nodules from thyroid cancer patients were mutation positive [138]. This result represents 100% diagnostic specificity. In FNABs with inconclusive reports via other molecular marker and morphological analyses, the TERT promoter mutation determination could be very helpful for an accurate definition of malignancy [119]. It has to be pointed out, however, that the prevalence of TERT promoter mutations is not high (10%–20%) in differentiated thyroid cancer, and the exclusion of malignancy should not be only based on undetectable TERT promoter mutations. The combined analysis of the mutant TERT promoter and other biomarkers is thus required to achieve a higher accuracy of thyroid nodule diagnosis.

In addition to the diagnostic potential of TERT promoter mutations in urological and thyroid cancer, they may serve as useful biomarkers in other malignancies. We are currently testing the mutant TERT promoter in blood from patients with hepatocellular carcinoma and other malignancies. Increased applications of the TERT promoter mutation as biomarkers for cancer diagnostics and disease surveillance will be expected in near future.

## 5. TERT Promoter Mutations: Novel Prognostic Factors in Cancer Patients

More and more studies have demonstrated a relevance of TERT promoter mutations with clinic-pathological characteristics in malignancies. We found that the presence of TERT promoter mutations was significantly associated with metastases in thyroid and renal pelvic carcinomas [136]. In thyroid FNAB analyses by Liu et al. [138], 80% of the TERT promoter mutation-positive thyroid cancers exhibited aggressive clinic-pathological behaviors, including extrathyroidal invasion, lymph node metastases, distant metastases, and disease recurrence. A number of additional investigations revealed a significant correlation between the presence of TERT promoter mutations and relapse in thyroid cancer [84,97,119]. A close relationship between the mutation and recurrence was also observed in other types of cancer [76]. Interestingly, the frequency of the TERT promoter mutation increased in advanced or progressive cancer [89,93,98,138]. It was documented that a significant increase in TERT promoter mutations occurred from well-differentiated PTC and FTC to anaplastic thyroid cancer (ATC), the most aggressive thyroid cancer [93]. In gliomas, the mutation frequencies were 39% and 76% in low and high grades of tumors, respectively [60]. TERT promoter mutations are evidently selected for cancer progression or invasion.

Direct evaluation of the TERT promoter mutation as a prognostic factor has been made in many types of cancer [87]. Thyroid cancer and glioma are most extensively studied malignancies with consistent results. We [93,99] analyzed the effects of TERT promoter mutations, BRAF^V600E^ and age (cutoff 45 years) on survival of patients with thyroid cancer, and our results showed that the TERT promoter mutation was the only variable that independently predicted shorter disease-related survival (DRS) in PTC; the presence of TERT promoter mutations together with age > 45 was significantly associated with DRS in FTC. There was no relationship between BRAF^V600E^ and patient survival in PTC. A similar finding was reported by George et al. and others [90,96]. However, Xing et al. analyzed 507 PTC patients and observed the co-presence of TERT promoter and BRAF^V600E^ as a powerful predictor for DRS [100]. In addition, it was shown that patients with the mutant TERT promoter-tumors had a significantly lower response rate to radioiodine therapy [83].

The relationship of TERT promoter mutations with patient outcomes in gliomas is revealed in most clinical studies [139], but more complex, and affected by other genetic alterations, TERT promoter polymorphism and tumor grades. Patients who had grade II and III gliomas with only TERT promoter mutations had poorer overall survival, however, those with co-existence of both TERT and IDH mutations exhibited favorable outcomes [60,65]. The mutation was unable to independently predict patient survival in grade IV gliomas [60]. Labussiere et al. studied 807 patients with glioma, and found that the presence of −124C>T or −146C>T mutations was associated with a significantly shorter overall survival (OS) in grade III and IV gliomas [140]. However, in sharp contrast, OS was longer for low-grade gliomas with the mutation. Another clinical report showed that −124C>T or −146C>T mutations were associated with poor OS in grade IV gliomas, but the effect was confined to the patients who did not carry the variant G-allele for the rs2853669 polymorphism at the TERT promoter [69,141].

The prognostic value of the TERT promoter mutation was also tested in other human malignancies. In a cohort of 327 patients with BC, Rachakonda et al. [82] showed an overall tendency of poor survival in the patients that carried the mutations in tumors. They further observed a phenomenon similar to that seen in gliomas: the TERT promoter polymorphism rs2853669, acting as a modifier of the effect of the TERT promoter mutation on survival and the mutation was significantly correlated with patient poor survival in the absence but not in the presence of the variant allele of the polymorphism. However, no association was found between the presence of the mutation and outcome in another analysis of 468 BC patients [77]. The TERT promoter mutation was an independent prognostic factor and correlated with a shorter DRS and OS in ovarian clear cell carcinomas, as documented by Huang et al. [143], while the other study did not show such correlation [144]. In patients with nonacral cutaneous and spitzoid melanomas, the TERT promoter mutation was independently associated with poorer OS [50,53], whereas Nagore et al. failed to show the mutation as an independent prognostic factor in patients with primary melanoma [120]. It is evident from the above results, the effect of the TERT promoter mutation on patient survival remains inconclusive for BC, ovary cancer, melanoma and many other malignancies, which calls for further clinical evaluations.

To better understand the relationship between TERT promoter mutations and patient outcomes, it is also important to dissect how this genetic event affects cancer cell behaviors and contributes to cancer progression. TERT or telomerase not only provides cancer cells with a proliferation advantage by stabilizing telomere size, but also displays multiple activities independently of telomere-lengthening function. TERT directly promotes cancer cell invasion and metastasis by inducing epithelial-mesenchymal transition and other mechanisms [148,149]. Consistently, Wu et al. showed that BC cells with either −124C>T or −146C>T mutations acquired enhanced cellular motility [132]. In addition, TERT was further shown to protect cancer cells from apoptosis stimulated by various insults or stresses [150,151,152,153,154,155,156,157,158,159]. Taken together, the TERT promoter mutation enhances high TERT expression, thereby contributing to aggressive phenotypes of cancer cells and poor patient outcomes via both telomere lengthening-dependent and independent mechanisms.

## 6. Perspectives

TERT and telomerase play a critical role in carcinogenesis, and thus elucidation of their regulatory mechanisms is highly demanding. The recent identification of TERT promoter mutations has significantly contributed to our understandings of telomerase activation in human malignancies. However, a number of important questions and challenges remain: How does the mutation occur and why does the frequency vary so much among different types of malignancies? What is the relationship between the mutant promoter and many other TERT signaling cascades and regulators, and is targeting the mutant promoter feasible and sufficient for telomerase inhibition in cancer cells? Importantly, is introducing the mutant allele alone capable of conferring differentiated cells an immortal phenotype? In addition, the TERT promoter mutation detection has demonstrated usefulness in cancer diagnostics and outcome prediction, but the obtained data are still preliminary and inconclusive, and more large-scale validation studies are definitely required. Further efforts to solve all the above issues will certainly promote new development and application of TERT-based diagnostics and managements in human cancer, thereby contributing to precision oncology.

## Figures and Tables

**Table 1:** A Summary of TERT promoter mutations in human cancer.

Tumor Type	Mutation Rate (%)	References
**Skin tumor**		
Base cell carcinoma	132/278 (47.4)	[112,113]
Squamous cell carcinoma	75/125 (60.0)	[112,113,114,135]
Merkel cell carcinoma	5/49 (10.2)	[127]
pleomorphic dermal sarcoma	26/34 (76.0)	[108]
Atypical fibroxanthoma	25/27 (93.0)	[108]
Malignant melanoma		
Cutaneous melanoma	564/1287 (43.8)	[5,6,48,49,50,51,54,57]
Other types of melanoma	165/505 (32.7)	[52,55]
**Brain tumor**		
Glioma (low-grade)	929/2580 (36,0)	[57,59,62,66,67,73]
Glioma (high-grade)	2171/3085 (70.4)	[57,59,62,66,67,69,70,71,72,79]
Meningioma	25/337 (7.4)	[75,76]
Medulloblastoma	36/182 (19.8)	[7,66]
**Endocrine tumor**		
Thyroid cancer		
Papillary thyroid carcinoma	593/5380 (11.0)	[57,83,84,86,87,88,89,90,91,92,93,94,95,96,97,100,110]
Follicular thyroid carcinoma	59/346 (17.1)	[57,83,92,93,94,95,96,99]
Anaplastic thyroid carcinoma	93/237 (39.2)	[57,83,93,94,96,98]
Hurthle cell carcinoma	8/61 (13.1)	[85]
Atypical follicular thyroid adenoma	3/18 (16.7)	[99]
Differentiated thyroid carcinoma	41/339 (12.1)	[94]
Poorly differentiated thyroid carcinoma	73/170 (42.9)	[57,94,96,110]
Adrenocortical carcinoma	4/98 (4.1)	[57,92]
**Gynecological tumor**		
Ovarian clear cell carcinoma	48/301 (15.9)	[7,143,144]
Ovarian low grade serous	2/41 (4.9)	[7,144]
Endometrial carcinoma	5/76 (6.6)	[7,143,144]
Squamous cell carcinoma of the cervix	33/335 (9.9)	[7,131,135,144]
**Urological tumor**		
Renal cell carcinoma	22/318 (6.9)	[57,79,122]
Bladder cancer	946/1511 (62.6)	[7,57,77,78,79,80,81,115]
Upper tract urothelial carcinomas		
Renal pelvic carcinoma	51/117 (43.6)	[7,136]
Ureter carcinoma	23/122 (19)	[136]
**Digestive system tumor**		
Hepatocellular carcinoma	363/881 (41.2)	[7,61,101,103,111]
Gastric cancer	0/200 (0)	[128]
**Head and neck tumor**		
Laryngeal carcinoma	64/235 (27.2)	[145]
Squamous cell carcinoma of head and neck	14/86 (16.3)	[7,135]
**Soft tissue and pleuron tumor**		
Myxoid liposarcoma	50/72 (69.4)	[7,107,109]
Solitary fibrous tumour	14/58 (24.1)	[7,66,107,109]
Chondrosarcoma	1/2 (50)	[7]
Fibrosarcoma	1/3 (33.3)	[7]
Malignant pleural mesothelioma	20/132 (15.2)	[104]
**Other tumors**		
Mantle cell lymphoma	8/24 (33.3)	[106]
Phyllodes tumor	30/46 (65.0)	[105]
Prostate cancer	108/167 (64.7)	[129]
Medullary carcinoma	0/62 (0)	[83,93,94]
pheochromocytoma	1/105 (1)	[92]
Paraganglioma	1/13 (7.7)	[92]
